# Fractal analysis of bone architecture changes following transcrestal sinus lifting with simultaneous implant placement

**DOI:** 10.1186/s12903-026-08816-3

**Published:** 2026-06-08

**Authors:** Mert Keles, Ibrahim Burak Yuksel

**Affiliations:** 1 Department of Periodontology, Faculty of Dentistry, Lokman Hekim University, Ankara, Turkey; 2 Department of Oral and Maxillofacial Radiology, Faculty of Dentistry, Necmettin Erbakan University, Konya, Turkey; 3 Present Address: Dental Clinic Academy, Ankara, 06510 Turkey

**Keywords:** Dental implants, Fractal analysis, Fractal dimension, Panoramic radiography, Trabecular bone, Transcrestal sinus lifting

## Abstract

**Background:**

Evidence regarding the microarchitectural characteristics of newly formed bone after transcrestal sinus lifting remains limited. This retrospective longitudinal study aimed to quantitatively evaluate trabecular microarchitectural changes in peri-implant bone using fractal analysis in patients who underwent transcrestal sinus lifting with simultaneous implant placement.

**Methods:**

Clinical and radiographic records of 76 patients treated with transcrestal sinus lifting and simultaneous implant placement in the posterior maxilla were retrospectively reviewed. Baseline and approximately 6-month follow-up panoramic radiographs were analyzed. Fractal analysis was performed on predefined regions of interest (ROIs) representing mesial and distal peri-implant trabecular bone, apical trabecular bone, and superior and inferior maxillary tuberosity trabecular bone.

The primary outcome was the change in peri-implant fractal dimension (FD) values over time. Secondary outcomes included changes in maxillary tuberosity FD values and inter-regional differences between peri-implant and tuberosity areas. Apical FD values were assessed as exploratory outcomes.

**Results:**

Peri-implant FD values increased significantly from baseline to 6 months (1.182 ± 0.054 vs. 1.233 ± 0.058; *p* < 0.001; Cohen’s d = 1.28). In contrast, median FD values in the maxillary tuberosity region remained stable over time (1.252 [1.215–1.286] vs. 1.256 [1.212–1.289]; *p* = 0.116; *r* = 0.18). At baseline, peri-implant FD values were significantly lower than those of the maxillary tuberosity region (1.180 [1.154–1.222] vs. 1.252 [1.215–1.286]; *p* < 0.001; *r* = 0.83), and although this difference remained significant at 6 months (1.249 [1.193–1.272] vs. 1.256 [1.212–1.289]; *p* < 0.001), the effect size decreased (*r* = 0.62). Exploratory analysis also showed a significant increase in median apical FD values (1.182 [1.145–1.225] vs. 1.253 [1.220–1.289]; *p* < 0.001; *r* = 0.84).

**Conclusions:**

In patients undergoing transcrestal sinus lifting with simultaneous implant placement, peri-implant FD values increased during the 6-month follow-up period, while FD values in the maxillary tuberosity reference region remained stable. These findings suggest that fractal analysis may provide a reproducible adjunctive radiographic method for monitoring trabecular pattern changes after transcrestal sinus lifting. However, FD changes should not be interpreted as direct evidence of histological bone maturation or implant stability without further clinical validation.

**Supplementary Information:**

The online version contains supplementary material available at 10.1186/s12903-026-08816-3.

## Introduction

Tooth loss is a common clinical condition that adversely affects individuals’ quality of life in both functional and esthetic terms. In addition to esthetic impairment, tooth loss may lead to compromised masticatory function, disruption of occlusal balance, and progressive resorption of the alveolar bone [1].

Maxillary molars are among the teeth most frequently lost, particularly due to periodontal reasons [2].

In the posterior maxilla, maxillary sinus pneumatization and the limited height of the residual alveolar bone, especially following the loss of the first and second molars, represent the main anatomic factors complicating implant therapy. Insufficient bone height may hinder the achievement of adequate primary stability during implant placement and, consequently, may negatively affect the success of osseointegration [3].

Maxillary sinus augmentation techniques, developed to overcome these anatomic and biomechanical limitations in the posterior maxilla, are widely used to improve the predictability of implant-supported prosthetic rehabilitation [4]. The primary aim of these procedures is to create sufficient bone height for implant placement through elevation of the sinus membrane. From a technical perspective, maxillary sinus lift procedures are generally classified into two main approaches: the lateral window approach and the transcrestal approach. While the lateral window technique is more commonly preferred in cases with a residual bone height of less than 4 mm, the transcrestal sinus lift technique has gained increasing acceptance in clinical practice because of its lower invasiveness, reduced morbidity, and the possibility of being performed simultaneously with implant placement [5]. Although several surgical modifications of the transcrestal sinus lift approach have been described, many studies have shown that the osteotome-mediated technique provides reliable long-term implant outcomes [6, 7]. In addition, this technique allows implant placement to be performed during the same surgical session in most cases, thereby shortening treatment time and reducing patient morbidity [8].

The long-term success of implant therapy depends not only on achieving adequate bone volume, but also on the microarchitectural characteristics and trabecular organization of the regenerated bone [9].

For this reason, the structural properties of peri-implant bone tissue should be evaluated using objective and quantitative methods. However, conventional radiographic assessments primarily focus on macroscopic parameters, such as bone height and density, and may be limited in detecting microstructural changes occurring within the trabecular bone architecture [10].

In this context, fractal analysis (FA) has emerged as an effective method for the quantitative assessment of the geometric complexity of trabecular bone structure on digital radiographic images [11]. In recent years, FA has been increasingly used to investigate the temporal reorganization of trabecular bone changes around dental implants [12]. This method allows the microarchitectural characteristics of newly formed peri-implant bone to be evaluated in a non-invasive and reproducible manner.

Although studies investigating trabecular bone changes around dental implants using FA have been reported in the literature, longitudinal evidence evaluating peri-implant bone architecture in patients undergoing transcrestal sinus lifting with simultaneous implant placement remains limited [13, 14].

Moreover, the extent to which the bone formed after transcrestal sinus lifting resembles native maxillary trabecular bone in structural terms has not yet been clearly established.

The aim of this study was to quantitatively evaluate trabecular microarchitectural changes in peri-implant bone tissue in patients undergoing transcrestal sinus lifting with simultaneous implant placement using FA. We hypothesized that fractal dimension (FD) values in the peri-implant bone would increase significantly over time after transcrestal sinus lifting and that this increase would reflect reorganization within the peri-implant trabecular bone. The distinctive aspect of this study is that it evaluates peri-implant trabecular changes after transcrestal sinus lifting not only over time, but also in comparison with a reference trabecular bone area unaffected by surgery.

## Materials and methods

### Study design, ethical approval, and patient selection

This retrospective longitudinal observational study was approved by the Lokman Hekim University Scientific Research Ethics Committee, Ankara, Turkey (Decision No: 2025/190; meeting date: 27 June 2025). The study was based exclusively on anonymized retrospective clinical and radiographic records obtained during routine patient care. No additional intervention, radiographic examination, or patient contact was performed for research purposes. Therefore, informed consent to participate was not obtained. All procedures were conducted in accordance with the principles of the Declaration of Helsinki, and the study was designed and reported in line with the STROBE (Strengthening the Reporting of Observational Studies in Epidemiology) guidelines. Clinical trial number: not applicable.

The study dataset was retrospectively compiled from the institutional archive using the clinical and radiographic records of patients who underwent transcrestal sinus lifting with simultaneous dental implant placement in the premolar and molar regions of the maxilla between 2021 and 2025. All radiographs included in the analysis were obtained from the patients’ routine clinical follow-up records.

Before the analysis, patient records were organized electronically by an independent researcher who was not involved in the study team. Each patient was assigned a unique identification number generated randomly in Microsoft Excel for Microsoft 365 (Microsoft Corporation, Redmond, WA, USA) using the RAND function. The list linking patient identities to analysis data was stored on a password-protected computer accessible only to the data manager. Thus, data anonymity was preserved by preventing the investigators from accessing patient-identifying information during the analysis process.

The inclusion criteria were age ≥ 18 years, simultaneous implant placement using the transcrestal sinus lifting technique in the posterior maxilla, and availability of baseline and approximately 6-month follow-up panoramic radiographs related to the implant procedure. The approximately 6-month follow-up period was selected because it corresponded to the routine healing interval before prosthetic loading in the study center for implants placed simultaneously with transcrestal sinus lifting. The exclusion criteria were the presence of systemic diseases that could affect bone metabolism, use of medications that could affect bone metabolism (e.g., bisphosphonates or corticosteroids), a history of surgical intervention in the maxillary tuberosity region within the previous 2 years, and radiographs with insufficient image quality or marked distortion that precluded diagnostic evaluation.

A total of 76 implants from 76 patients were evaluated in the study. In cases where a patient had more than one implant, the implant to be included in the analysis was determined by simple random selection using the RAND function in Microsoft Excel. This approach was adopted to reduce the clustering effect and statistical dependence that could arise from including multiple implants from the same patient.

### Surgical procedure

Information regarding the surgical procedures was retrospectively obtained from patient files and clinical records. According to the clinical records, all surgical procedures were performed under local anesthesia by an experienced periodontist (MK) using the osteotome-mediated transcrestal sinus lifting technique.

Following incision and mucoperiosteal flap elevation, implant osteotomy was prepared according to the manufacturer’s surgical protocol. The sinus floor was then carefully elevated using osteotomes, and Schneiderian membrane elevation was achieved. Implants were placed during the same surgical session without the use of grafting material. All implants were manufactured by Osstem Implant Co. (Seoul, Korea); implant length was 8.5 mm in all cases, and diameters ranged from 4.0 to 4.5 mm [15].

During the postoperative period, all patients received a standard medication protocol. According to the clinical records, patients were prescribed amoxicillin–clavulanic acid, ibuprofen, and a mouth rinse containing 0.12% chlorhexidine gluconate. Review of the follow-up records indicated that no intraoperative or postoperative complications were reported after the surgical procedure.

### Radiographic imaging protocol

All panoramic radiographs were obtained using the same panoramic device (Planmeca ProOne^®^, Planmeca Oy, Helsinki, Finland). To ensure standardization, all imaging procedures were performed by a single trained radiology technician, and fixed exposure parameters were used for all patients (68 kVp, 7 mA, 10 s). The use of the same device, the same acquisition protocol, and constant exposure settings was intended to minimize imaging-related variability. Because all images were acquired using a single imaging system, additional standardization across different panoramic devices was not required. The raw panoramic images were exported and analyzed in their original digital format without resizing, resampling, interpolation, or AI-based super-resolution processing.

The images were evaluated using Planmeca Romexis^®^ software (Planmeca Oy, Helsinki, Finland); brightness, contrast, and filter settings were kept constant across all images. All radiographic evaluations were performed using the same image-processing protocol and under standardized viewing conditions.

All radiographic analyses were carried out on a calibrated monitor (24-inch Full HD monitor, 1920 × 1080 pixels, 120 Hz; Dell UltraSharp U2415). The analyses were performed under constant ambient lighting conditions.

### Region of interest (ROI) selection

To evaluate trabecular bone architecture by FA, all measurements were performed using ImageJ software (version 1.53; National Institutes of Health, Bethesda, MD, USA).

For each implant site, regions of interest (ROIs) measuring 30 × 30 pixels were manually selected on the panoramic radiographs. This ROI size was based on a standardized measurement commonly used in previous FA studies and considered suitable for the reliable assessment of trabecular bone architecture on panoramic radiographs [13].

The 30 × 30 pixel ROI size was kept constant for all images and anatomical regions. ROI dimensions were not adjusted according to implant position or premolar/molar location. If a 30 × 30 pixel ROI could not be placed entirely within trabecular bone without including cortical bone, implant surfaces, radiographic artifacts, or major anatomical superimposition, that region was not used for measurement.

All ROI selections were performed by a single observer blinded to patient-identifying information. The observer had access only to the anonymized dataset containing randomly generated patient identification numbers. To reduce potential temporal bias, the images were evaluated in random order.

During ROI selection, care was taken to include only trabecular bone tissue. Cortical bone structures, implant surfaces, radiographic artifacts, and areas of major anatomical superimposition were excluded at the ROI selection stage; these structures were not included within the rectangular ROI and were not subtracted after ROI placement.

**Fig. 1:**
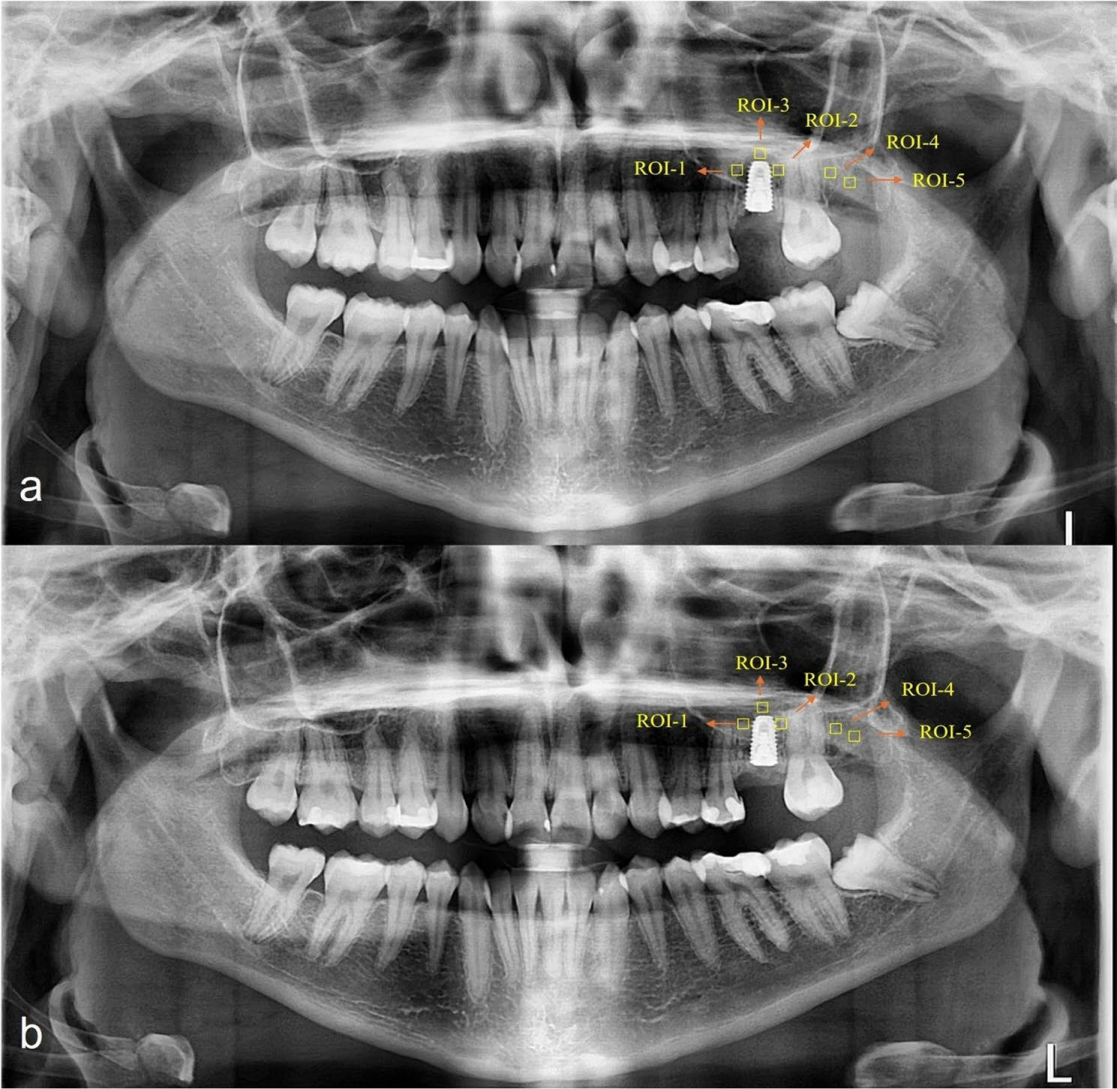
Representative panoramic radiographs illustrating the regions of interest used for fractal analysis after transcrestal sinus lifting with simultaneous implant placement. **A** Baseline image obtained immediately after surgery. **B** Six-month follow-up image. ROI-1 and ROI-2 correspond to the mesial and distal peri-implant trabecular bone regions, respectively; ROI-3 corresponds to the apical trabecular bone region immediately below the implant apex; and ROI-4 and ROI-5 correspond to the superior and inferior trabecular bone regions of the maxillary tuberosity, respectively, which served as reference regions

The selected ROI regions (Fig. 1) were defined as follows:

- ROI-1: peri-implant trabecular bone located mesial to the implant
- ROI-2: peri-implant trabecular bone located distal to the implant
- ROI-3: apical trabecular bone located immediately below the implant apex
- ROI-4: trabecular bone located in the superior portion of the maxillary tuberosity
- ROI-5: trabecular bone located in the inferior portion of the maxillary tuberosity

ROI-1, ROI-2, and ROI-3 were selected within the surgically modified peri-implant region associated with transcrestal sinus lifting and simultaneous implant placement. Because no grafting material was used, these ROIs represented peri-implant trabecular bone within the elevated sinus/implant region rather than grafted bone. ROI-4 and ROI-5 were selected from pristine maxillary tuberosity trabecular bone and served as reference ROIs. In anatomically complex regions, such as areas potentially affected by third molars, marked tuberosity slope, cortical overlap, or major superimposition, ROIs were placed only when sufficient trabecular bone was available for a 30 × 30 pixel measurement area; otherwise, the region was not used for measurement. 

### Image preprocessing and fractal analysis

**Fig. 2:**
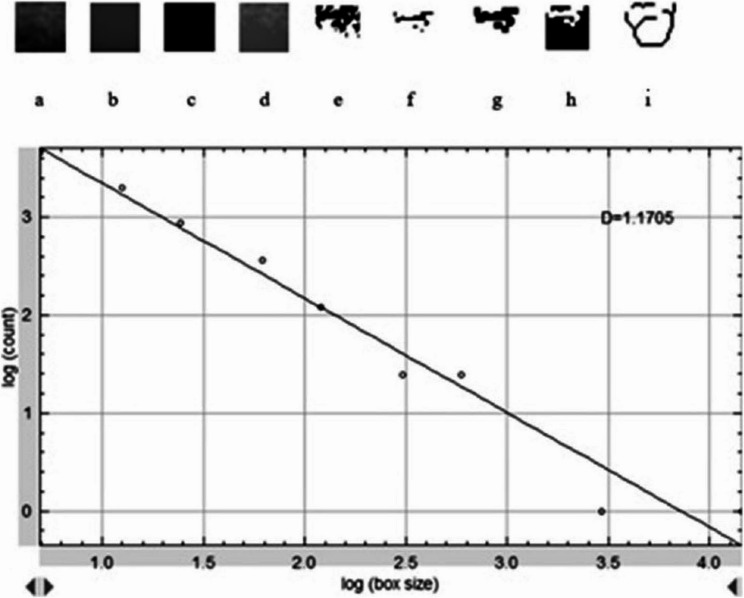
Image preprocessing workflow and fractal dimension calculation used for fractal analysis. Representative ImageJ-based preprocessing workflow for fractal analysis. **a** Original ROI image, (**b)** Gaussian-blurred image, (**c**) subtracted image, (**d**) normalized image after addition of a gray value of 128, (**e**) binarized image, (**f**) eroded image, (**g**) dilated image, (**h**) skeletonized image, and (**i**) box-counting output with the corresponding log–log plot used for fractal dimension (FD) calculation. FD was obtained from the slope of the regression line relating box count to box size

Before FA, all ROI images were processed using a standardized image-processing protocol in ImageJ and exported in TIFF format (Fig. 2). The predefined ROIs were cropped and prepared for analysis. No AI-based enhancement, super-resolution processing, or separate noise-reduction algorithm was applied before Gaussian blurring or fractal analysis. Panoramic artifacts were controlled primarily at the image selection and ROI selection stages by excluding radiographs with insufficient image quality or marked distortion and by avoiding artifact-contaminated regions during ROI placement. The images were then sequentially converted to 8-bit grayscale, subjected to Gaussian blur filtering (σ = 35 pixels), and the blurred image was subtracted from the original image. This subtraction step was part of the standard fractal analysis preprocessing workflow and was not used to remove cortical bone, implant surfaces, artifacts, or superimposed anatomical structures from within the ROI. Gaussian blurring was used as part of the standard box-counting fractal analysis workflow rather than as an independent image-enhancement procedure. A gray value of 128 was subsequently added to normalize image intensity. The images were then binarized using a global thresholding method, followed by erosion and dilation procedures to enhance the trabecular pattern. In the next step, the images were skeletonized to reduce the trabecular structures to their linear representations.

FD was calculated using the “Fractal Box Count” function in ImageJ. For this purpose, the skeletonized ROI images were overlaid with square grids of 2, 3, 4, 6, 8, 12, 16, 32, and 64 pixels. The number of occupied boxes was recorded for each box size, and the slope of the regression line obtained from the log–log plot of box count versus box size was accepted as the FD value.

The peri-implant FD was calculated as the mean of ROI-1 and ROI-2. The tuberosity FD was determined as the mean of ROI-4 and ROI-5. The apical FD was calculated from ROI-3.

### Measurement reliability

To assess intraobserver reliability of the FD, 20 implant sites were randomly selected from the dataset for repeated analysis (RAND function, Microsoft Excel). This number was determined to represent at least 20% of the total sample. The second measurement was performed 2 weeks later, and the observer was blinded to the results of the first measurement. Agreement between the two measurements was evaluated using the intraclass correlation coefficient (ICC), calculated with a two-way mixed-effects model (absolute agreement), and 95% confidence intervals were reported.

### Statistical analysis

All statistical analyses were performed using IBM SPSS Statistics for Windows, Version 27.0 (IBM Corp., Armonk, NY, USA). The sample size was calculated using G*Power software (version 3.1; Heinrich-Heine-Universität Düsseldorf, Germany). Based on an assumed medium effect size (effect size = 0.50), a 95% confidence level (α = 0.05), and 80% statistical power (power = 0.80), the minimum required sample size was determined to be 34 implants. The 76 implants evaluated in the present study exceeded this minimum requirement.

Continuous variables were summarized as mean ± standard deviation (SD). For variables that did not meet normality assumptions, median and interquartile range values were additionally reported to improve transparency. Normality of data distribution was assessed using the Shapiro–Wilk test, together with visual inspection of histograms and Q–Q plots.

The primary outcome of the study was the comparison of peri-implant FD values (mean of ROI-1 and ROI-2) between baseline and the 6-month radiographs. Secondary outcomes included the temporal changes in maxillary tuberosity FD values (mean of ROI-4 and ROI-5) and the differences in FD values between the peri-implant and tuberosity regions. Apical FD values (ROI-3) were evaluated as part of the exploratory analysis.

For longitudinal comparisons of baseline and 6-month FD values from the same implant region, the paired-samples t-test was used when the distribution of paired differences met normality assumptions, whereas the Wilcoxon signed-rank test was used when these assumptions were not met. Comparisons between the peri-implant and maxillary tuberosity regions at the same time point were also performed using either the paired-samples t-test or the Wilcoxon signed-rank test, according to the distribution of paired differences. To assess the magnitude of observed differences, effect size was calculated using Cohen’s d for parametric analyses and r for nonparametric analyses. As an exploratory subgroup analysis, peri-implant FD change was compared between smokers and non-smokers. Peri-implant FD change was calculated as the difference between the 6-month and baseline peri-implant FD values.

Depending on data distribution, the independent-samples t-test or Mann–Whitney U test was used for this comparison.

Because the primary, secondary, and exploratory outcomes were defined before analysis, no additional Bonferroni correction was applied for multiple comparisons. Statistical significance was set at *p* < 0.05.

## Results

**Fig. 3:**
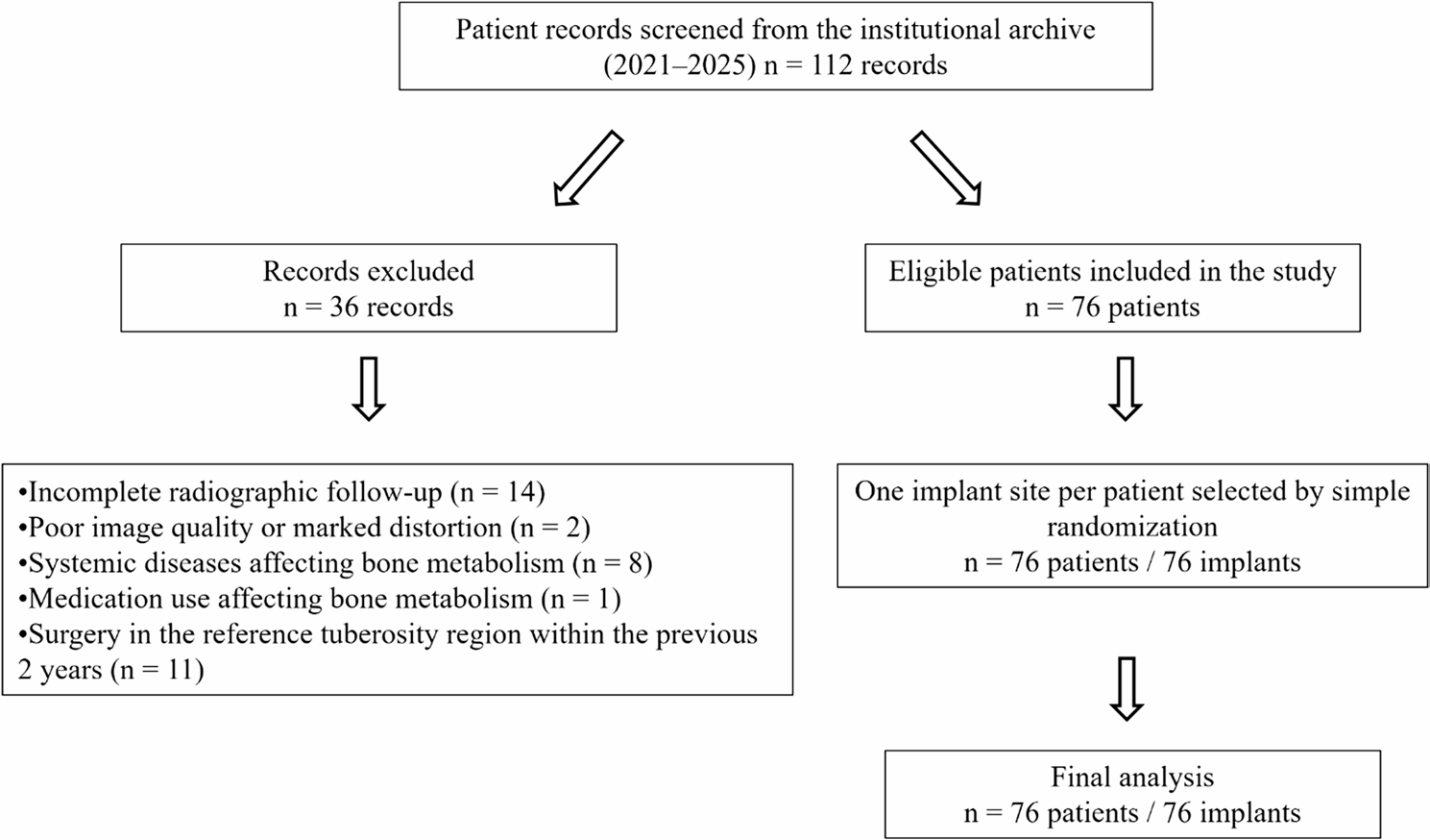
Study flow diagram showing record screening, exclusions, eligibility assessment, and final inclusion of 76 patients and 76 implants in the analysis

A total of 112 patient records were initially screened. Of these, 36 records were excluded because of incomplete radiographic follow-up (*n* = 14), poor image quality or marked distortion (*n* = 2), systemic disease affecting bone metabolism (*n* = 8), medication affecting bone metabolism (*n* = 1), or surgery in the maxillary tuberosity region within the previous 2 years (*n* = 11). After application of the predefined inclusion and exclusion criteria, 76 eligible patients were included in the study. One implant site per patient was selected for analysis, resulting in a final dataset of 76 patients and 76 implants (Fig. 3).

The mean age of the patients was 48.1 ± 8.5 years; 48 patients were male (63.2%) and 28 were female (36.8%). In the study group, 29 patients (38.2%) were recorded as smokers and 47 (61.8%) as non-smokers (Table 1).

**Table 1:** Demographic characteristics of the study sample (*n* = 76 patients)

Variable	Value
Age (years), mean ± SD	48.1 ± 8.5
Sex, male, n (%)	48 (63.2%)
Sex, female, n (%)	28 (36.8%)
Smoking, yes, n (%)	29 (38.2%)
Smoking, no, n (%)	47 (61.8%)

Data are presented as mean ± SD or n (%)

### Primary outcome: peri-implant fractal analysis findings

FD values representing the peri-implant trabecular bone architecture (mean of ROI-1 and ROI-2) were 1.182 ± 0.054 at baseline and 1.233 ± 0.058 at the 6-month follow-up. Median and interquartile range values are presented in Table 2. According to the paired-samples t-test, this increase was statistically significant (*p* < 0.001). The effect size calculated for this change was large (Cohen’s d = 1.28) (Table 2).

**Table 2:** Longitudinal changes in fractal dimension values between baseline and 6 months (*n* = 76 patients/implants)

Region / Measure	Baseline, mean ± SD	Baseline, median [Q1–Q3]	6 months, mean ± SD	6 months, median [Q1–Q3]	Test used	*p* value	Effect size
Peri-implant FD	1.182 ± 0.054	1.180 [1.154–1.222]	1.233 ± 0.058	1.249 [1.193–1.272]	Paired-samples t-test	< 0.001	Cohen’s d = 1.28
Tuberosity FD	1.242 ± 0.055	1.252 [1.215–1.286]	1.243 ± 0.054	1.256 [1.212–1.289]	Wilcoxon signed-rank test	0.116	*r* = 0.18
Apical FD	1.190 ± 0.058	1.182 [1.145–1.225]	1.248 ± 0.051	1.253 [1.220–1.289]	Wilcoxon signed-rank test	< 0.001	*r* = 0.84

Data are presented as mean ± SD and median [Q1–Q3]. Peri-implant FD was calculated as the mean of ROI-1 and ROI-2, tuberosity FD as the mean of ROI-4 and ROI-5, and apical FD from ROI-3. The paired-samples t-test was used when the distribution of paired differences met normality assumptions, whereas the Wilcoxon signed-rank test was used when these assumptions were not met. Effect size is presented as Cohen’s d for parametric analyses and r for non-parametric analyses

*FD* Fractal dimension, *IQR* Interquartile range, *Q1* First quartile, *Q3*, Third quartile, *SD* Standard deviation.

### Secondary outcomes

#### Maxillary tuberosity fractal analysis findings

FD values for the maxillary tuberosity region, used as the reference trabecular area (mean of ROI-4 and ROI-5), were 1.252 [1.215–1.286] at baseline and 1.256 [1.212–1.289] at the 6-month follow-up. According to the Wilcoxon signed-rank test, this change was not statistically significant (*p* = 0.116). The effect size was small (*r* = 0.18) (Table 2).

#### Inter-regional comparisons

At baseline, peri-implant FD values were significantly lower than those of the maxillary tuberosity region (1.180 [1.154–1.222] vs. 1.252 [1.215–1.286], *p* < 0.001). The effect size for this comparison was large (*r* = 0.83). At 6 months, peri-implant FD values remained significantly lower than those of the maxillary tuberosity region (1.249 [1.193–1.272] vs. 1.256 [1.212–1.289], *p* < 0.001), although the effect size was smaller (*r* = 0.62) (Table 3).

**Table 3:** Inter-regional comparison of peri-implant and maxillary tuberosity fractal dimension values at baseline and 6 months (*n* = 76 patients/implants)

Time point	Peri-implant FD (mean ± SD)	Peri-implant FD, median [Q1–Q3]	Tuberosity FD (mean ± SD)	Tuberosity FD, median [Q1–Q3]	Test used	*p* value	Effect size
Baseline	1.182 ± 0.054	1.180 [1.154–1.222]	1.242 ± 0.055	1.252 [1.215–1.286]	Wilcoxon signed-rank test	< 0.001	*r* = 0.83
6 months	1.233 ± 0.058	1.249 [1.193–1.272]	1.243 ± 0.054	1.256 [1.212–1.289]	Wilcoxon signed-rank test	< 0.001	*r* = 0.62

Data are presented as mean ± SD and median [Q1–Q3]. Peri-implant FD was calculated as the mean of ROI-1 and ROI-2, and tuberosity FD as the mean of ROI-4 and ROI-5. Comparisons between peri-implant and tuberosity FD values at each time point were performed using the Wilcoxon signed-rank test. Effect size is presented as r

*FD* Fractal dimension, *IQR* Interquartile range, *Q1* First quartile, *Q3* Third quartile, *SD* Standard deviation

#### Exploratory outcomes: apical fractal analysis findings

FD values for the apical trabecular bone region (ROI-3) were 1.182 [1.145–1.225] at baseline and 1.253 [1.220–1.289] at the 6-month follow-up. According to the Wilcoxon signed-rank test, this increase was statistically significant (*p* < 0.001). The effect size calculated for the exploratory analysis was large (*r* = 0.84) (Table 2).

#### Exploratory subgroup analysis according to smoking status

The mean peri-implant FD change was 0.056 ± 0.037 in smokers and 0.049 ± 0.043 in non-smokers. Median [Q1–Q3] values were 0.056 [0.039–0.078] and 0.047 [0.020–0.069], respectively. According to the independent-samples t-test, peri-implant FD change did not differ significantly between smokers and non-smokers (*p* = 0.431; Cohen’s d = 0.19).

#### Measurement reliability

Intraobserver reliability analysis showed good to excellent agreement for the FD measurements. ICC values ranged from 0.815 to 0.991. The highest agreement was observed for the tuberosity and peri-implant measurements, while apical measurements also demonstrated good reliability (Supplementary Material S1).

## Discussion

In this retrospective longitudinal study, changes in peri-implant bone architecture were quantitatively evaluated by FA in patients who underwent transcrestal sinus lifting with simultaneous implant placement. The increase in peri-implant FD may indicate radiographic trabecular pattern reorganization around the implant after transcrestal sinus lifting (from 1.182 ± 0.054 to 1.233 ± 0.058; *p* < 0.001; Cohen’s d = 1.28), rather than direct histological evidence of bone maturation. An FD increase of approximately 0.05–0.06 represents a subtle but measurable increase in radiographic trabecular complexity. Clinically, this may suggest progressive trabecular reorganization within the peri-implant region during the early healing period. However, this magnitude should not be interpreted as a validated clinical threshold for successful bone regeneration, implant stability, or implant prognosis. This finding is in line with previous studies reporting radiographic bone formation and bone gain and may be regarded as an indirect radiographic indicator of trabecular reorganization [7, 16]. In contrast, the absence of a significant change in the maxillary tuberosity region (1.252 [1.215–1.286] at baseline and 1.256 [1.212–1.289] at 6 months; *p* = 0.116; *r* = 0.18) suggests that the observed trabecular change was localized to the surgical area. In addition, the finding that the peri-implant region showed lower FD values than the maxillary tuberosity bone at baseline, with a reduction in this difference at 6 months (baseline: 1.180 [1.154–1.222] vs. 1.252 [1.215–1.286], *p* < 0.001, *r* = 0.83; 6 months: 1.249 [1.193–1.272] vs. 1.256 [1.212–1.289], *p* < 0.001, *r* = 0.62), suggests that despite the persistence of statistical significance, the degree of separation decreased over time and that the observed peri-implant change may be more closely related to local healing and reorganization than to general temporal variation.

The increase in FD observed in the apical region, evaluated as part of the exploratory analysis, is also noteworthy (1.182 [1.145–1.225] at baseline and 1.253 [1.220–1.289] at 6 months; *p* < 0.001; *r* = 0.84); however, these findings should be interpreted with greater caution. In FA, ROI selection is a critical methodological step that directly affects the results, and anatomic variations may limit the reliability of standardized ROI placement in this region [17]. Therefore, the apical data should be considered supportive of the primary findings but interpreted as exploratory in nature.

In the current literature, studies on transcrestal/crestal sinus lifting have largely focused on clinical outcomes such as surgical techniques, implant survival, primary stability, bone gain, and marginal bone loss [18, 19]. In contrast, data on the microarchitectural characteristics and bone quality of the newly formed bone after transcrestal sinus lifting remain limited. Although some studies have reported favorable outcomes regarding bone gain following this procedure [20, 21], Dhore et al. noted that the results related to bone gain after transcrestal sinus lifting are heterogeneous and that further research is needed in this field [6]. Therefore, it is important to evaluate the bone formed after transcrestal sinus lifting not only quantitatively but also in terms of its structural characteristics.

A large part of the FA literature has focused on cases treated with the lateral window approach and grafting procedures, with most studies examining graft maturation, comparisons of different graft materials, and radiographic changes following the lateral window technique [14, 22]. In contrast, data on the evaluation of peri-implant trabecular changes in cases undergoing transcrestal sinus lifting with simultaneous implant placement remain very limited. To the best of our knowledge, the only study in this context compared the transcrestal and lateral window approaches [13]. Therefore, a direct one-to-one comparison with the present results is not possible; however, that study provides indirect support for our findings by demonstrating that FA can be used to assess trabecular bone changes after sinus lifting. The present study, on the other hand, offers a distinctive contribution to this limited body of literature by focusing exclusively on cases treated with transcrestal sinus lifting and by using a reference trabecular region unaffected by surgery.

FA is a non-invasive method capable of quantitatively evaluating changes in trabecular bone organization [23]. Its applicability to dental radiographs, the possibility of serial assessment without additional radiation exposure, and its relatively low cost make it a practical option for clinical research [24, 25]. In the literature, while some studies have reported that cone-beam computed tomography (CBCT) may provide a more detailed assessment than panoramic radiography in the context of FA [26], other studies have suggested that panoramic radiography may be the more suitable option for evaluating trabecular bone structure, given that this difference is not always pronounced and that panoramic imaging offers advantages such as wider availability and faster acquisition [27, 28]. These discrepancies suggest that imaging modality, reconstruction parameters, and ROI selection may all influence FD results. In addition, it should be recognized that FA results may be affected by image quality, ROI selection, image-processing steps, and the algorithm used [17]. Therefore, FA should be regarded not as a substitute for histologic examination or three-dimensional imaging methods, but rather as a complementary radiographic indicator that may assist in monitoring trabecular reorganization.

An important clinical aspect of this study is that FA offers a non-invasive, accessible, and quantitative means of assessment using routine panoramic radiographs. In clinical settings where advanced imaging methods are not always necessary or readily available, this approach may serve as a useful adjunct for the serial monitoring of trabecular reorganization around implants. However, it should also be recognized that the prognostic value of FD changes in clinical decision-making has not yet been directly established. Therefore, if supported by future evidence of prognostic validity, FD changes may potentially contribute to monitoring the healing response after sinus lifting and to the complementary radiographic assessment of bone response before prosthetic loading. Nevertheless, FA findings should not be considered directly equivalent to histologic bone quality, but rather as an indirect radiographic indicator of trabecular organization. Furthermore, the present study focused on radiographic trabecular pattern changes, and FD values were not correlated with clinical stability parameters such as implant stability quotient values. Therefore, the observed FD increase should not be interpreted as direct evidence of improved implant stability.

This study has several important strengths. Owing to its longitudinal design, trabecular bone changes in the same implant region were evaluated at two time points, and the peri-implant findings were compared with those of the tuberosity region, which served as a reference trabecular area unaffected by surgery.

In addition, including only one implant per patient reduced statistical dependence. The use of the same radiographic device and fixed exposure parameters for all images, blinded analyses, a standardized ROI size, and confirmation of intraobserver reliability by ICC further support the methodological robustness of the study. In particular, the use of the maxillary tuberosity as a reference trabecular region provided an important methodological advantage in interpreting the local nature of the changes observed in the peri-implant area.

However, the present study also has several limitations. Due to its retrospective design, dependence on record quality and the possibility of selection bias could not be completely eliminated. Smoking may represent a potential confounder because of its known influence on bone healing and implant-related outcomes. Although an exploratory subgroup analysis was performed and no statistically significant difference in peri-implant FD change was observed between smokers and non-smokers, the present study was not primarily designed or powered to evaluate the independent effect of smoking on FD changes. Therefore, smoking should still be considered a potential confounder, and the subgroup findings should be interpreted cautiously. In addition, the two-dimensional nature of panoramic radiographs entails limitations such as magnification, geometric distortion, and anatomical superimposition; therefore, this method cannot fully represent the three-dimensional microarchitecture of bone. Panoramic distortion and superimposition may influence FD values by altering the apparent density, continuity, and spatial distribution of trabecular structures. In the posterior maxilla, overlap from the maxillary sinus floor, zygomatic buttress, tuberosity cortex, and adjacent roots may obscure or artificially modify the trabecular pattern. Although the use of the same device, fixed exposure parameters, standardized ROI selection, and exclusion of poor-quality images reduced this risk, residual distortion-related measurement variability cannot be fully eliminated. The follow-up period was limited to 6 months, which precluded evaluation of longer-term trabecular pattern changes and post-loading outcomes. The lack of histologic validation is another limitation. Nevertheless, in this clinical scenario involving simultaneous implant placement with transcrestal sinus lifting, obtaining a biopsy for research purposes would not be ethically or surgically appropriate; therefore, bone reorganization was assessed at the radiographic level. Furthermore, the relationship between FD changes and implant stability, prosthetic loading success, or long-term clinical outcomes was not directly evaluated in this study.

Finally, the single-center design may limit the generalizability of the findings.

Future studies with longer follow-up periods, larger sample sizes, and designs supported, whenever feasible, by CBCT and/or histologic correlation would be valuable. In addition, investigating the effects of different surgical protocols, implant designs, and graft use on FA findings in transcrestal sinus lifting may further expand the clinical knowledge base in this field. Furthermore, validation of image-processing procedures and ROI standardization for FA across different centers could strengthen both the comparability and the clinical applicability of the method.

## Conclusion

Within the limitations of this retrospective radiographic study, peri-implant FD values increased significantly during the 6-month follow-up period in patients undergoing transcrestal sinus lifting with simultaneous implant placement, while the difference between the peri-implant region and the maxillary tuberosity reference area decreased. These findings suggest that FA may serve as a reproducible adjunctive radiographic method for monitoring trabecular pattern changes after transcrestal sinus lifting.

However, the observed FD increase should not be interpreted as direct evidence of histological bone maturation, bone quality, or implant stability without further validation using clinical, histological, or long-term outcome measures.

## Supplementary Information

Supplementary Material 1.

## Data Availability

De-identified datasets and analysis files supporting the findings of this study are available from the corresponding author upon reasonable request.

## Abbreviations

CBCT: Cone-beam computed tomography
FA: Fractal analysis
FD: Fractal dimension
ICC: Intraclass correlation coefficient
ROI: Region of interest
SD: Standard deviation
STROBE: Strengthening the Reporting of Observational Studies in Epidemiology
