# Supplementary material for: Fractal analysis of bone architecture changes following transcrestal sinus lifting with simultaneous implant placement
Source: BMC Oral Health. 2026 Jun 8;26:1425. doi: 10.1186/s12903-026-08816-3 (PMC13465137; doi:10.1186/s12903-026-08816-3)
Supplement: Supplementary file 1 [file 12903_2026_8816_MOESM1_ESM.docx]

**Supplementary Table S1.** **Intraobserver reliability of fractal measurements assessed using the intraclass correlation coefficient (ICC)**

| Measurement region | ICC (3,1) | 95% Confidence Interval (CI) | Reliability level | p value |
| --- | --- | --- | --- | --- |
| Periimplant Baseline | 0.959 | [0.899 – 0.983] | Excellent | < 0.001 |
| Periimplant 6 Month | 0.990 | [0.974 – 0.996] | Excellent | < 0.001 |
| Tuberosity Baseline | 0.991 | [0.978 – 0.996] | Excellent | < 0.001 |
| Tuberosity 6 Month | 0.984 | [0.959 – 0.993] | Excellent | < 0.001 |
| Apikal Baseline | 0.815 | [0.590 – 0.922] | Good | < 0.001 |
| Apikal 6 Month | 0.853 | [0.666 – 0.939] | Good | < 0.001 |

ICC (3,1) values are presented with 95% confidence intervals (CI). Intraobserver agreement was assessed using a two-way mixed-effects model with absolute agreement. Reliability was interpreted as follows: <0.50, poor; 0.50–0.75, moderate; 0.75–0.90, good; and >0.90, excellent. p < 0.05 was considered statistically significant.
